# Incidence of Febrile Seizures in Children with COVID-19

**DOI:** 10.3390/jcm12031076

**Published:** 2023-01-30

**Authors:** Min Jeong Han, Jun Ho Heo, Ji Seong Hwang, Young-Taek Jang, Min Lee, Sun Jun Kim

**Affiliations:** 1 Department of Pediatrics, Jeonbuk National University Hospital, Jeonju 54907, Republic of Korea; 2 Research Institute of Clinical Medicine, Pediatrics, Medical School, Jeonbuk National University, Jeonju 54907, Republic of Korea; 3 Biomedical Research Institute, Jeonbuk National University Hospital, Medical School, Jeonju 54907, Republic of Korea; 4 Department of Emergency Medicine, Wonkwang National University, Iksan 54538, Republic of Korea; 5 Department of Pediatrics, Jeonbuk Gunsan Medical Center, Gunsan-si 54105, Republic of Korea; 6 Department of Pediatrics, Presbyterian Medical Center, Jeonju 56075, Republic of Korea; 7 Department of Pediatrics, Design Medical Center, Jeonju 54910, Republic of Korea

**Keywords:** children, coronavirus disease 19, febrile seizure, SARS-CoV-2

## Abstract

The coronavirus disease 2019 (COVID-19) has become a common cause of febrile seizures (FS), especially after the Omicron surge. This study aimed to determine the incidence of COVID-19-associated FS in children. The number of confirmed COVID-19 cases in patients aged below five years residing in the Jeonbuk province from January 2020 to June 2022 was obtained from official data provided by the Ministry of Public Administration and Security. During the same period, data on FS patients with COVID-19 were obtained from all local hospitals capable of FS treatment and were analyzed retrospectively. The number of children under five years of age in Jeonbuk was 62,772, of which 33,457 (53.2%) were diagnosed with COVID-19 during the study period. Of these, 476 patients (1.4%) required hospitalization, and 64 (0.19%, 44 boys; 68.8%: 20 girls; 31.2%) developed FS. All patients with FS presented with symptoms after the Omicron surge. Before the Omicron variant, 23.4% of the patients (89 of 381) required hospitalization; however, no children with COVID-19 were hospitalized for FS. Twenty-five patients (39.1%) had complex FS while one (1.6%) presented with febrile status epilepticus. Forty-two patients (65.6%) experienced first-time FS with an average of 1.5 convulsive events.

## 1. Introduction

Febrile seizures (FS), which is a common neurologic disorder in children aged 6–60 months, are convulsions that happen when a child has a fever but does not have any intracranial infection. Approximately 2–5% of children reportedly experience FS [1]. FS are further classified into three distinct categories: simple FS, complex FS (CFS), and febrile status epilepticus (FSE). Simple FS are generalized seizures that occur only once in a 24 h period and last less than 15 min. CFS can be defined as seizures with a focal onset that occurs more than once during a 24 h period or that last more than 15 min [2,3]. Generally, FS are known to not be associated with negative effects on future intellect or behavior; however, they are sometimes reported to be associated with a higher rate of neurodevelopmental problems [4,5]. Although the exact etiology of FS is unclear, it occurs frequently during fever episodes caused by various vaccinations, as well as during viral and bacterial infections [6,7,8]. The consensus on the viruses that most commonly cause FS varies depending on the published data; however, adenoviruses, influenza, human herpesvirus-6, and rhinoviruses are reported to be the main culprits [9,10,11]. Thus far, studies on these viruses have been focused on the incidence rate and clinical characteristics of FS among hospitalized patients for each virus. Reportedly, 15–20% of patients hospitalized for influenza A infection develop FS [12]. However, since these studies have focused on hospitalized patients who are relatively difficult to manage, the reported rates do not reflect the actual prevalence of FS in the general population of people with confirmed viral infections. Moreover, the novel coronavirus SARS-CoV-2 (coronavirus disease 2019; COVID-19) has an advantage when it comes to estimating the prevalence rate of FS over the total population of patients, since the overall incidence of COVID-19 is determined in the general population as a national statistic.

The COVID-19 pandemic, which began in 2020, changed the incidence rates of several diseases [13,14,15]. Owing to the valiant efforts of the Korean government during the COVID-19 pandemic, the COVID-19 infection rate in children was minimal during the early stages of the pandemic. As of 1 July 2022, the ratio of the cumulative number of confirmed cases of COVID-19 to the population of South Korea stood at 37.9%, which ranked 8th worldwide [16]. Byeon et al. reported that the prevalence of other viral infections and the incidence of FS in South Korea were significantly reduced as mask-wearing and strict personal hygiene were implemented during the COVID-19 pandemic [17]. However, the incidence of FS during COVID-19 has not yet been reported, except for a paper published in 2022 in the United States that reported its incidence at 0.5% [18]. This study also aimed to determine the number of FS cases before the Omicron surge, and we predicted that the incidence of FS among COVID-19 confirmed cases would increase after the appearance of Omicron. In addition, this study aimed to predict the incidence of FS after COVID-19 by calculating the ratio of the number of FS to the number of COVID-19 cases among children under the age of 5 years and to investigate the clinical characteristics of these children.

## 2. Methods

### 2.1. Patients

Jeonbuk is a province in South Korea with a population of 1.78 million people and 62,772 children under 5 years of age. Five hospitals (one in each of the three most densely populated cities in the province) in this province can provide medical care for FS. As a province with a limited number of general and tertiary hospitals, Jeonbuk has favorable conditions for the study of the incidence rate of specific diseases, as most patients with FS receive their final treatment in these hospitals. Retrospective data were collected from children diagnosed with COVID-19 under 60 months of age in Jeonbuk province from January 2020, when the first COVID-19 case was reported in Korea, to June 2022, after the Omicron variant pandemic. Five hospitals in each of the three most populous cities were dedicated to the treatment of children with COVID-19. The number of patients with confirmed COVID-19 and those with FS aged between 6 months and 5 years. Most Koreans can undergo COVID-19 polymerase chain reaction (PCR) testing with only minor symptoms or a history of contact with a COVID-19 patient. Patients with confirmed COVID-19 were quarantined for seven days, and those requiring medical treatment due to high fever, seizure, or respiratory distress were admitted to the hospital. These recommendations were also applicable to children. All participants tested positive on COVID-19 PCR tests after hospitalization.

We included children under 5 years of age with confirmed COVID-19 who presented convulsions with fever. The International League against Epilepsy defines FS as seizures that occur as a result of a high-grade fever (over 38 °C) with the absence of central nervous system infection, inflammation, or an acute systemic metabolic abnormality that may induce convulsions [19]. According to the abovementioned criteria, among convulsive patients, those who had underlying epilepsy, no fever, or other causes of convulsions, such as electrolyte imbalances and structural anomalies in brain magnetic resonance imaging (MRI), were excluded from the FS group.

### 2.2. Data Collection

We investigated the number of children under the age of 5 years who were diagnosed with COVID-19 in Jeonbuk province during the study period. The number of confirmed COVID-19 cases was obtained from COVID-19-related public data provided by the Ministry of Public Administration and Security. In the Republic of Korea, since 7 April 2021, all public health centers perform PCR tests for COVID-19 diagnosis for patients without suspicious symptoms or epidemiological associations.

The charts of COVID-19-positive patients in five hospitals were reviewed to identify all patients with symptoms of FS. To determine the incidence of FS associated with COVID-19, we divided the number of patients with FS due to COVID-19 infection by the total number of COVID-19 confirmed children. Additionally, patient characteristics, including demographic characteristics, laboratory findings, type of seizures, duration, electroencephalography, and length of hospitalization, were also evaluated. Each patient’s laboratory data were obtained on the day of admission. To determine the effects of COVID-19 on the severity of FS, the characteristics of children with prolonged FS (PFS) were also recorded.

Using SPSS Version 23.0 for windows, the mean and standard deviation were calculated for continuous variables, such as age, number of seizures, and duration, while categorical variables, such as sex and seizure type, were summarized using frequencies and percentages. The Institutional Review Board (IRB No. CUH 2022-06-001-006 and WKUH 2022-06-015) of our center approved the research proposal.

## 3. Results

### 3.1. Study Population

**Figure 1:**
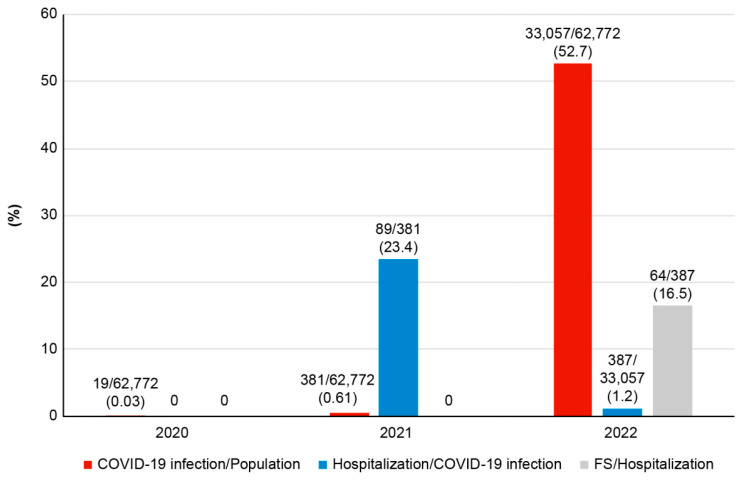
Confirmed COVID-19 cases, hospitalization rates, and febrile seizures according to sex.

**Figure 2:**
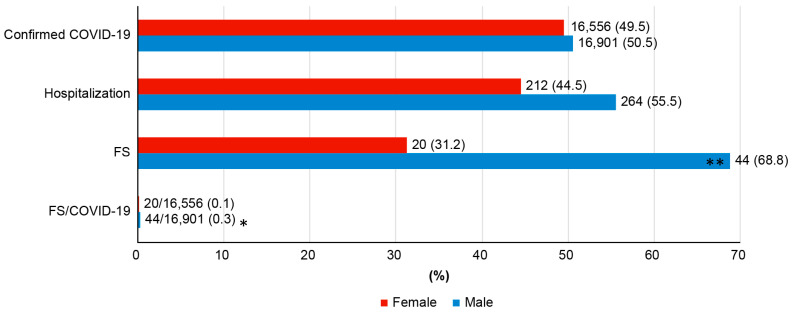
Incidence of confirmed COVID-19 cases, febrile seizures, and hospitalization rates according to year during the COVID-19 pandemic. The * indicates the percentage of boys with febrile seizures among those with confirmed COVID-19. The ** indicates the percentage of boys among those who showed febrile seizures.

During the study period, 53.2% (33,457 of 62,772) of children aged less than five years in Jeonbuk were confirmed to have COVID-19. Of these 33,457 patients, the number of patients with COVID-19 was 19 (0.03%) in 2020, 381 (0.6%) in 2021, and 33,057 (52.6%) by June 2022 (Figure 1); of the confirmed patients, 476 (1.4%) were boys, and 264 (girls: 212) required hospitalization (Table 1); their mean age was 26.9 ± 18.3 months (1–60 months) (Table 1). Of the 476 hospitalized patients, 71 had convulsions with fever; however, 7 of them had already been diagnosed with epilepsy and were prescribed anticonvulsant medications (ASMs). Finally, 64 patients (0.19%); (44 boys, 68.8%; 20 girls, 31.2%) were diagnosed with COVID-19-associated FS (Figure 2, Table 1). The ratio of FS to confirmed COVID-19 cases according to sex was 0.3% for male patients and 0.1% for female patients (Figure 2). FS cases were first observed in 2022 after the Omicron variant surge (Figure 1). The mean age of children with FS was 36.7 ± 15.0 months.

### 3.2. Clinical Manifestations in Children with COVID-19-Associated FS

Twenty-five patients (39.1%) with FS had CFS, and only one patient (1.6%) presented with FSE, which was defined as a seizure activity lasting at least 30 min [20]. Forty-two patients (65.6%) experienced first-time FS, and the remaining 22 patients (34.4%) had a history of FS (Table 2). Children with FS had an average of 1.5 ± 1.2 convulsive episodes, and the patient with the most convulsions experienced ten episodes in three days. The median duration of FS was 3.0 min, and the longest reported seizure lasted for 60 min and required ventilator care. Thirteen children (20.3%) had a family history of FS in first-degree relatives, and the mean hospitalization period was 3.7 ± 2.7 days. Most patients did not need ASM after hospitalization; however, seven patients (10.9%) required primary ASM (intravenous [IV] lorazepam, 0.1 mg/kg). Furthermore, two of these patients required secondary ASM (IV fosphenytoin, 20 mg/kg), and one patient required a continuous intravenous infusion of midazolam (5 µg/kg/min). Most of the patients had normal leukocyte counts (7.49 ± 2.11 × 10^3^/µL) and lymphocyte counts (22.5% ± 13.2%), and the median serum C-reactive protein level was 1.71 mg/L (range, 0.2–36).

### 3.3. Neurologic Manifestations of Patients with Prolonged FS during COVID-19

We studied the characteristics of six patients with PFS lasting more than 15 min [21]. Table 3 shows that most of the patients with PFS were boys (n = 5, 83.3%). Four patients (66.7%) experienced their first seizures due to COVID-19. Electroencephalography (EEG) and brain MRI were performed for four patients (66.7%), while the other two children could not undergo additional examinations during quarantine. All four patients showed no abnormalities in their brain MRIs, and three out of four showed slow background activity in their EEGs; however, no electrical epileptiform discharge was observed. Despite the long seizure duration in all patients, hospital stays lasted less than 8 days, and only two patients (33.3%) required ASM. The glucose levels of those who required ASM were 232 mg/dL and 291 mg/dL, and their lactate levels were 3 mmol/L and 26 mmol/L, respectively. The convulsive events in other patients were self-limiting.

## 4. Discussion

We observed a significant increase in the number of patients with FS from December 2021, when the Omicron variant was first reported in Korea (Table 1) [22]. Until 2021, prior variants of COVID-19 had been associated with severe disease and a 20.5% hospitalization rate among the infected patients; however, no FS was observed (Figure 1, Table 1). Conversely, after the Omicron surge, 16.5% of hospitalized patients (64 of 387, Figure 1) had FS, despite the hospitalization rate dropping to 1.2% (387 of 33,057). This result concurs with the findings of a study conducted in South Africa, which reported that 20% of hospitalized patients aged below 19 years with the Omicron variant experienced seizures [23]. Human herpesvirus-6 or the influenza A virus, which are common causes of FS, also led to a 10–20% incidence of FS in hospitalized patients [24,25]. Thus, this study provides more evidence that the Omicron variant can also be a common cause of FS.

The mean age of patients with FS due to COVID-19, 36.7 ± 15.0 months, was higher than the peak age of FS, which is 18–20 months [26,27]. Furthermore, 39.1% of patients with FS had CFS, and 1.6% of them had FSE, which is also higher than the average 25–30% incidence of CFS [28,29]. In 2022, Apirada et al. reported the characteristics of 16 pediatric patients with seizures due to COVID-19 after the Omicron surge [30]. Six patients (38%) presented with focal seizures and eight patients (50%) presented with status epilepticus. However, a higher incidence of status epilepticus than that found in our study was reported per the criteria for status epilepticus (seizures lasting more than 5 min) [28]. Eleven children (17.2%) had seizures lasting more than 5 min in our study, which also indicated that neurological symptoms occurred more frequently than in the previous COVID-19 variants.

Overall, this study confirmed that the Omicron variant could also be a common cause of FS, and CFS and FSE occurred more frequently at an older age. The findings of this study may be due to the increase in the total number of confirmed COVID-19 cases; however, they suggest the possibility of the Omicron variant leading to poor neurologic clinical outcomes, even though the disease severity is less than that associated with the previous variants. The mechanism underlying the invasion of the central nervous system in patients with COVID-19 has been introduced in many studies, and it seems to be associated with neuronal inflammation mediated by various cytokines [31,32]. Furthermore, according to the genomic epidemiology of the SARS-CoV-2 Omicron variants, these variants exhibit enhanced transmissibility despite the widespread population immunity when compared with prior variants [33]. Although the causes of the neuroinvasive character of Omicron variants have not been clarified, we suggest that the potential higher infectivity of these variants is associated with the incidence of FS. However, the neuropathogenesis of COVID-19 remains unclear.

The biggest advantage of this study was that the incidence of FS among confirmed COVID-19 patients could be calculated based on government statistical records rather than inpatient population organizations. Only 0.19% of children with COVID-19 developed FS. This can be considered an advantage of previous COVID-19 research, in which inpatients were the target population [34,35]. To the best of our knowledge, the existing reports on COVID-19-related FS were also studies on hospitalized patients [18,20]. Our study may contribute to strengthening the basis for predicting the incidence of FS in the general COVID-19 population. It is thought that the number of FS cases among children under the age of 5 years can be predicted. Additionally, we found that the incidence of FS was more than doubled in boys compared to girls. Previous studies before the Omicron surge suggested lower incidence values of FS among Korean children [17], and the possibility that the incidence of FS may increase after the Omicron pandemic should not be neglected.

In this study, we investigated all hospitals where FS treatment was available in Jeonbuk province. However, one of the limitations is that the precise incidence may differ because of the loss of patients who received medical care at primary hospitals and those who did not receive treatment at all. Additionally, selection bias may have occurred, because the studies were conducted in only one province in Korea. Nationwide research is needed to estimate the incidence rate of FS more accurately by comparing the number of patients diagnosed with FS and COVID-19 simultaneously in all hospitals across the country.

## 5. Conclusions

FS, which may occur in approximately 0.19% of all COVID-19 patients under the age of five years, appears to have been more frequent with the Omicron variant in boys. Moreover, CFS and FSE were more likely to occur with this variant.

## Figures and Tables

**Table 1:** Demographic features of children with coronavirus 2019 in Jeonbuk.

	COVID-19 Infection	Hospitalized COVID-19 (% *)	FS with COVID-19 (% **)
Number (n)			
2020		0	0
2021		89 (23.4)	0
2022		387 (1.2)	64 (0.19)
Total		476 (1.4)	64 (0.19)
Sex distribution (M:F)		0	
2020	9:10	0	0
2021	186:195	47:42	0
2022	16,706:16,351	217:170	44:20
Total	16,901:16,556	264:212	44:20
Age (months)			
2021		31.2 ± 16.7	
2022		25.8 ± 18.6	36.7 ± 15.0 (6–60)
Total		26.9 ± 18.3	

COVID-19; coronavirus 2019, FS; Febrile seizure. The * indicates the ratio of hospitalized patients with COVID-19 to confirmed COVID-19 patients. The ** indicates the ratio of febrile seizure patients with COVID-19 to confirmed COVID-19 patients.

**Table 2:** Clinical manifestations in children with COVID-19-associated febrile seizures.

Type of FS, n (%)	
Simple	39 (60.9)
Complex	24 (37.5)
FSE	1 (1.6)
First FS, n (%)	42 (65.6)
Recurrent FS, n (%)	22 (34.4)
Average episode of seizure, n	1.5 ± 1.2
Duration of seizures, median, minutes	3 (range 0.5–60)
Family history of FS, n (%)	13 (20.3)
Hospitalization, day	3.7 ± 2.7
Ventilator care, n (%)	1 (1.6)
ASM, n (%)	
1st	7 (10.9)
2nd	2 (3.1)
3rd or more	1 (1.6)
WBC, ×10^3^/µL	7.49 ± 2.71
Differential lymphocyte, %	22.5 ± 13.2
CRP median, mg/L	1.71 (range 0.2–36)

FS; febrile seizure, FSE; Febrile status epilepticus, ASM; antiseizure medication, WBC; white blood cell, CRP; C-reactive protein.

**Table 3:** Clinical characteristics of prolonged febrile seizures with COVID-19.

	Sex	Age (Months)	Previous FS	Epi	Duration (min)	EEG	bMRI	Admission (Days)	ASM	Glucose (mg/dL)	Lactate (mmol/L)
Pt 1	M	13	0	1	60	Slow waves	n/s	5	2nd	232	3
Pt 2	M	7	0	2	20	-	-	8	1st	291	26
Pt 3	M	28	0	1	15	-	-	1	X	78	0.9
Pt 4	M	57	10	3	15	Slow waves	n/s	2	X	91	1
Pt 5	F	37	0	2	20	Slow waves	n/s	3	X	95	1.33
Pt 6	M	51	1	1	15	n/s	n/s	6	X	106	1.6

FS, febrile seizure; Epi, episode; EEG, electroencephalography; bMRI, brain magnetic resonance imaging; ASM, antiseizure medication; n/s, no specific findings; Pt, patient; X, no ASM.

## Data Availability

Not applicable.
